# Fabrication of Electrospun PVA/Zein/Gelatin Based Active Packaging for Quality Maintenance of Different Food Items

**DOI:** 10.3390/polym15112538

**Published:** 2023-05-31

**Authors:** Sana Ullah, Motahira Hashmi, Jian Shi, Ick Soo Kim

**Affiliations:** 1 Nano Fusion Technology Research Group, Interdisciplinary Cluster for Cutting Edge Technologies, Institute of Fiber Engineering (IFES), Shinshu University, Ueda Campus, Ueda 386-8567, Nagano, Japan; sanamalik269@gmail.com; 2 Institute of Inorganic Chemistry I, Helmholtz Institute of Ulm (HIU), Ulm University, Helmholtzstrasse 11, 89081 Ulm, Baden Württemberg, Germany; 3 Faculty of Textile Science and Technology, Shinshu University, Ueda 386-8567, Nagano, Japan; shi@shinshu-u.ac.jp

**Keywords:** active food packaging, antioxidant, zein, gelatin, shelf life

## Abstract

In this research, electrospun PVA/Zein/Gelatin based tri-component active food packaging has been fabricated to enhance the shelf life of food by assuring the food quality (freshness, taste, brittleness, color, etc.) for longer. Electrospinning imparts good morphological properties along with breathability in nanofibrous mats. Electrospun active food packaging has been characterized to investigate the morphological, thermal, mechanical, chemical, antibacterial and antioxidant properties. Results of all tests indicated that the PVA/Zein/Gelatin nanofiber sheet possessed good morphology, thermal stability, mechanical strength, good antibacterial properties along with excellent antioxidant properties, which makes it the most suitable food packaging for increasing the shelf life of different food items like sweet potatoes, potatoes and kimchi. Shelf life of sweet potatoes and potatoes was observed for a period of 50 days, and shelf life of the kimchi was observed for a period of 30 days. It was concluded that nanofibrous food packaging may enhance the shelf life of fruit and vegetables because of their better breathability and antioxidant properties.

## 1. Introduction

One third of the food produced in the world is wasted every year which accounts for almost 1.3 billion tons. Enough food is produced to feed all humans globally, however 9.9% of the population is still facing hunger around the world. Food safety with enhanced shelf life is a step to achieve the goal of zero hunger. Fabrication of a novel nanofiber based active food packaging is important to minimize the wastage of food by increasing the shelf life of food products. The main purpose of active food packaging is the preservation of food without compromising the quality of the products.

Electrospinning is the simple, diverse and unique spinning technique for the fabrication of nanofibers, and it has so many applications in different research fields like tissue engineering [1], masks [2,3], wound dressings [4,5,6,7,8,9,10,11], drug delivery [3,4], membranes [12], food packaging, textiles and electronics. Besides food packaging, masks, wound dressings, textiles, and electronics, nanofibers also perform well as a lubricant additive [13]. Electrospun food packaging materials are very common in the industry due to their versatile properties. Active food packaging is the rapidly emerging face of the packaging industry [14]. Active packaging helps to preserve the food for a long time without compromising the quality. It helps to keep the food fresh and also enhances the shelf life of fruits and vegetables. Several materials having biocompatibility, biodegradability, antioxidant and antimicrobial properties which find their place in this field.

Zein is a corn-based protein (from which is possesses about 80% of its protein) and it is well known due to its hydrophobicity, biocompatibility, biodegradability and film forming properties [15,16,17,18]. Presence of non-polar amino acids imparts water resistance in zein films but it also causes lower gas permeability. Zein is biopolymer which is a material most suitable for edible food packaging but it is brittle with poor mechanical properties. [19,20,21,22,23] Mechanical properties can be improved by the incorporation of PVA, which has good mechanical properties and flexibility. A hydroxyl group is present on the backbone chain of PVA which is the reason for inter and intra hydrogen bonding. The presence of hydrogen bonding imparts mechanical strength in it. PVA is water soluble, biocompatible, non-toxic, thermally and mechanically stable synthetic polymer [24,25,26]. Polyvinyl alcohol has been used with different bioactive materials for antibacterial applications, moisture absorbance, and enhanced mechanical properties [27,28,29].

Gelatin is a sustainable biopolymer commonly derived from animals. It is colorless, tasteless, biocompatible and biodegradable and it forms gum while mixing in water [15,17,18]. Triple helix contents are present on the chain of gelatin protein which improves the physical properties of gelatin. It is very difficult to electrospin gelatin due to its water resistance. Therefore, the crosslinking of gelatin is very important before electrospinning [15,17,18,21,30,31,32,33]. Glutaraldehyde is a commonly used cross linker which imparts the mechanical strength in gelatin and made it easily electrospinable [14,34]. Incorporation of zein in this solution helps to improve the water resistance and the presence of gelatin transmits physical properties in the final film while the presence of PVA enhances the mechanical and thermal properties [35,36]. Combination of PVA, zein and gelatin crosslinked by glutaraldehyde provides a suitable food packaging film which has good biocompatibility, biodegradability, antimicrobial, mechanical and thermal properties.

Electrospun composite nanofibrous mats with PVA, zein, and gelatin have been fabricated to acquire combined properties (described above with reference to the cited literature) of all the materials. Zein and gelatin are natural polymers which might be considered as a step towards sustainability, while PVA was chosen considering its easy electrospinnability for the bulk production for commercial food packaging. A number of studies have already investigated the feasibility and role of electrospun nanofibers for food packaging. Most of the literature includes an active material for imparting antioxidation or antibacterial properties on to nanofibers. A similar study was done using feather keratin/PVA/PEO nanofibers supported by silver nanoparticles [28]. In contrast with the literature, we did not use any bioactive agent/drug or nanoparticles to acquire antioxidation, but rather an inherently antioxidant polymer was selected. Considering the wide range of applications of all these three polymers and their nanofibers using the electrospinning technique, it is expected to create a novel nanofiber mat with excellent biodegradability, biocompatibility, antibacterial and antioxidant properties which makes it the most suitable active food packaging application.

## 2. Experimental Work

### 2.1. Materials

Polyvinyl Alcohol (PVA) in powder form with an average molecular weight of Mw 85,000–124,000 (87–89% hydrolyzed), zein powder with less than 8% water content, and ethanol (99.5% pure) were purchased from Sigma-Aldrich (Saint Louis, MO 63103, USA). Gelatin (lot number WEH1584, case number 077-03155) was purchased from Wako pure chemicals industries ltd., Tokyo, Japan.

### 2.2. Methods

Three solutions were prepared. Solution of zein was prepared in the ethanol and distilled water. Ethanol and distilled water were taken with the percentage of 3:1. 20 wt.% of zein was taken as solute and the first type of solution was prepared. The second solution was prepared with gelatin as the solute and acetic acid as the solvent. 3 wt.%, 4 wt.% and 5 wt.% of gelatin was taken and in the second category, solutions of three different wt.% were prepared. Solution of 10 wt.% PVA was prepared in distilled water. The solution of Zein, Gelatin and PVA was mixed with 1:1:2. The electrospinning setup (Fluidnatek LE-50, Bioinicia, Valencia, Spain) was used for the fabrication of nanofibers. The plastic syringe along with the metallic needle was used to feed the solution. The voltage of 15 kV was applied from a high voltage DC power supply. The distance of 15 cm was kept between the needle and collection drum with the flow rate of 1.5 mL/h. All spinning solutions were run on electrospinning for a time period of 12 h to get free-standing nanofibrous mats.

## 3. Characterizations

Morphological properties of zein/gelatin, PVA/gelatin and zein/PVA/gelatin nanofibers were investigated by Scanning Electron Microscope (SEM), JSM-5300, JEOL Ltd., Osaka, Japan, accelerated with 15 kV voltage. The average diameter of the nanofibers was calculated by taking 50 arbitrary readings by using Image J software (Version ij153). Antibacterial activity of samples was assessed by the disk diffusion method. *E. coli* and *B. subtilis* bacteria strains were associated for gram negative and gram-positive bacteria strains, respectively. Samples were cut in the form of round disks of 13 mm in diameter and were put in cultured plates for both types of bacteria strains. The incubation time was 12 h at a 37 °C temperature. The chemical reaction between zein/PVA/gelatin was scrutinized by FTIR (ATR Prestige-21, Shimadzu, Japan). ATR spectra were taken from the wavenumber of 600 cm^−1^ to 4000 cm^−1^. The crystal structure of the composite nanofibers was assessed by Wide angle X-ray diffractions (WA-XRD) spectra. The DPPH protocol was adopted to analyze the antioxidant activity. The reaction took place in darkness at 4 °C. After 2 h of reaction, absorbance at 512 nm was measured. Percentage inhibition (%I) was calculated by the comparison of the initial absorbance *Ai* and the absorbance after 2 h, which was named as the final absorbance *A_f_* by using the following equation.

(1) $$\%I=\frac{Ai-Af}{2Ai}\times 100$$

Thermal stability of zein/PVA/gelatin nanofibers was investigated by thermo-plus TG 8120, Rigaku Corporation, Osaka, Japan. Heating rate was maintained at 10 °C/min with the temperature range, from room temperature to 500 °C using aluminum pans. Water contact angle was determined by cutting the nanofibrous samples into 2 × 7 cm^2^ of a rectangle shape. Fully air-dried samples were placed on a microscope slide, and 3 µL of distilled water was dropped on the sample’s surface through a 23-gauge blunt needle tip. A photograph of each water droplet on the nanofibrous mat was acquired (after 3 s) and analyzed by Smartdrop (Femtobiomed, Seongnam, Republic of Korea). The average of the left and right angles was taken as the water contact angle. For each sample, five specimens were characterized and an average of five readings was considered as water contact of the specific nanofibrous sample.

The test was performed in static mode. Mechanical properties were examined by the universal testing machine (UTM), Tensilon RTC 250A, A&D Company Ltd., Tokyo, Japan. The ISO 13634 standard was followed to prepare samples and five samples of each model were prepared. Tests were performed at room temperature under a crosshead speed of 5 mm/min. Tensile Stress, tensile strain, and Young’s modulus were calculated by using UTM data through the following equations.

(2) $$\varepsilon =\frac{∆l}{l}$$

(3) $$\sigma =\frac{F}{A}$$

(4) $$E=\frac{\sigma }{\varepsilon }$$

where ε,σ, and E represent strain, stress, and Young’s modulus respectively. l is the original length of the sample where ∆l is the change in length, F represents the applied force, and A is the cross-sectional area. Rotaflex RT300 mA, Rigaku, Osaka, Japan, was used to analyze XRD spectra with the angular angle ranging 5 ≤ 2θ ≤ 80° at 25 °C. Nickel-filtered Cu was used to take measurements.

## 4. Results and Discussions

### 4.1. Morphological Properties

**Figure 1:**
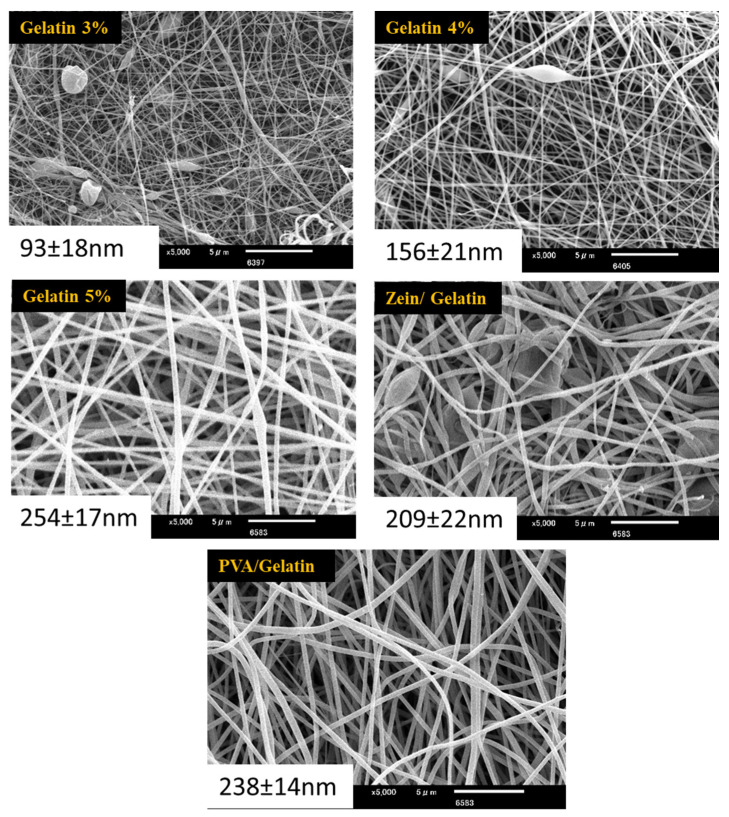
SEM images of PVA/Gelatin nanofibers, Zein/Gelatin nanofibers, and nanofibers of PVA/Zein/Gelatin with variable concentration of gelatin. The average diameter (nm) of nanofibers are given on each image.

Morphological properties were examined by SEM. Ratios of zein (20%) and PVA (10%) were kept constant while gelatin ratio fluctuated to get bead-free smooth nanofibers. SEM images of pure zein show beads which indicates the poor viscosity of zein. Addition of PVA and gelatin improved the viscosity of zein, but beads can still be seen in Figure 1. with the zein ratio 3%, which indicates that the ratio of gelatin is not enough to get the bead-free smooth nanofibers. As the ratio of gelatin increases, it imparts viscosity in the solution, which results in the bead-free smooth nanofibers with an increasing trend of the diameter. The image of 3% gelatin shows very thin nanofibers and an average diameter of the nanofibers of 93.71 nm.

Image with 5% gelatin shows the bead-free smooth nanofibers with an average diameter of 254 nm, which can be considered smooth and strong enough for food packaging application. It is also very difficult to electrospin simple gelatin. The addition of zein transmits a plasticizing effect and helps to decrease the interaction of gelatin molecules and improves the electrospinnability of gelatin. The increase in the diameter has been observed as the ratio of gelatin increases. The sample with the gelation ratio of 5% possessed the maximum average diameter of nanofibers.

### 4.2. X-ray Diffraction (XRD)

**Figure 2:**
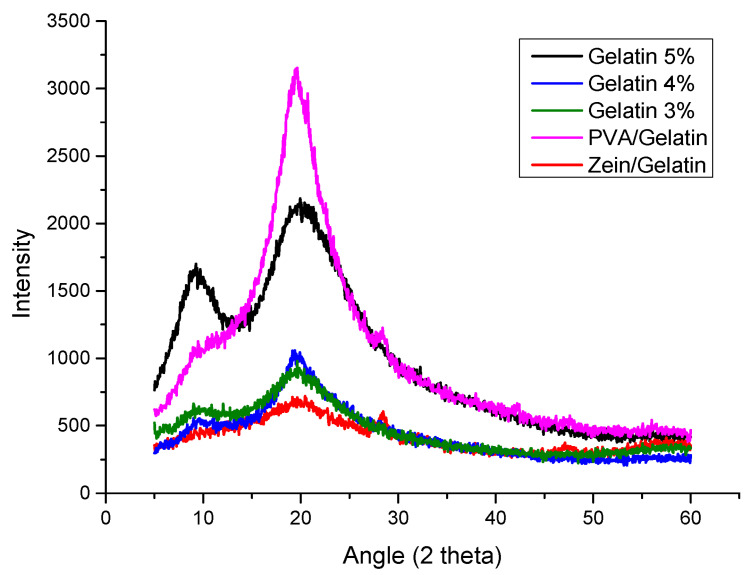
XRD of gelatin 5%, gelatin 4%, gelatin 3%, PVA/gelatin and Zein/gelatin.

**Figure 3:**
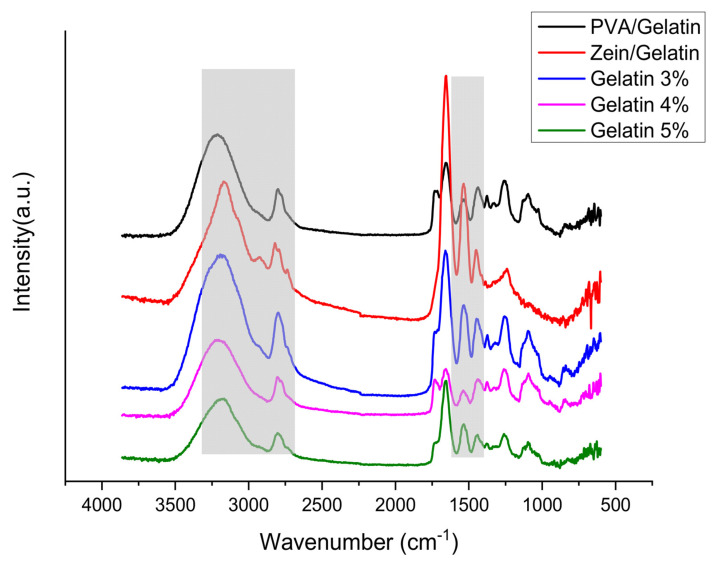
FTIR analysis of PVA/Zein/Gelatin nanofibers.

XRD analysis was performed to analyze the crystalline structure of individual components present in samples and also to observe the change in behavior with the addition of other components. To observe the crystalline structure the angle was taken at 2θ. In Figure 2, nanofibers of PVA/Gelatin show a sharp peak at 20° which represents the PVA and the presence of a small peak at around 28° represents the gelatin [37]. The sharp peak of PVA has been overcome with the addition of gelatin, however the sample with 5% gelatin showed the sharper peak than the samples with 4% and 3% gelatin. Decreasing the amount of gelatin showed the decrease in the peak. A sharp peak at 10° in the sample with 5% gelatin showed the presence of zein [24]. It can be observed from Figure 3 that the peak becomes wide with the decreasing amount of gelatin but the presence of the peak is an indication of the presence of zein. The Zein/Gelatin sample showed two peaks, one at 20° and another at 28°, the first represents the zein and the later shows the presence of gelatin. XRD analysis showed that the sample of 5% gelatin possessed good mechanical properties and can be used as a good food packaging material.

### 4.3. Fourier-Transform Infrared Spectroscopy (FTIR)

The possibility of chemical interactions among functional groups of zein, gelatin, and PVA was investigated by Fourier-transform infrared spectroscopy (FTIR) with an attenuated total reflection (ATR) assembly (Figure 3). Amide I and amide II functionalities were found common in all samples at wavenumbers of 1650 cm^−1^ and 1540 cm^−1^ respectively, representing a stretching vibration of C=O and C-N bonds, respectively. C-H stretching vibrations affiliated to aliphatic groups were found at a wavenumber of 2960 cm^−1^ for zein/gelatin samples. C-N stretching and N-H bending combination peaks were observed at a wavenumber of 1453 cm^−1^, C-N stretching was observed at 1406–1412 cm ^−1^, while the peak of deformation of the methyl group (C-H bong) was found at 1336 cm ^−1^, and absorption bands at 1245 cm ^−1^ were attributed to the combination of C-N stretching and N-H bending, respectively. N-H stretching vibrations of amide A were observed between 3400 cm^−1^ and 3250 cm^−1^, which was a broader peak as compared to others. The zein/gelatin sample is hydrophobic because of the strong intensity of O-H stretching in the FTIR wavenumber ~3250 cm^−1^. It is also noted that the sample with low O-H stretching intensity (Gelatin 5%) has a low contact angle. Uniform dispersion of PVA, zein, and gelatin within the fibers can be confirmed by there being no splitting of any of the peaks. There was no significant sequence in the intensity of the peaks depending upon variations in the concentration of gelatin in composite nanofibers. Additionally, no significant peak shift was observed which suggests there were no signs of chemical reaction among different functional groups, however there may be intermolecular or intermolecular bonding because of uniform dispersion.

### 4.4. Antioxidant Activity

**Figure 4:**
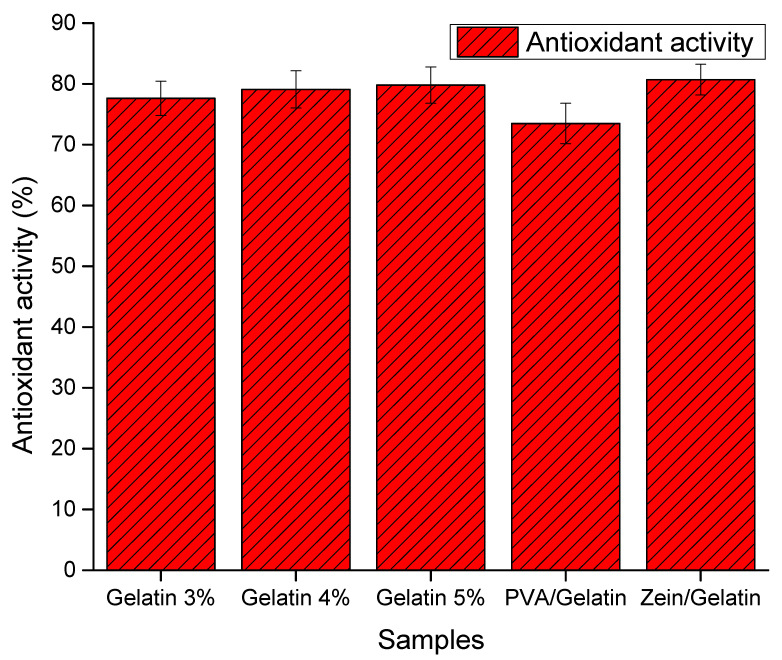
Antioxidant activity of PVA/Zein/Gelatin nanofibers.

Antioxidant activity of food packaging plays a vital role to extend the shelf life of food items without damaging the quality of food. The DPPH free radical test is one of the promising analyses to analyze the antioxidant activity with the help of free radical scavengers or a hydrogen donor. We analyze the samples with different ratios of gelatin from 3% to 5%, keeping the PVA and zein ratios same. Nanofiber sheets with PVA and Gelatin show the maximum percentage of inhibition rate, which indicates the maximum antioxidant activity. The Figure 4 presence of phenolic groups on PVA chains enhance the ability of the antioxidant activity. Zein does not contain any of the phenolic group which leads to the drop in antioxidant activity. Gelatin and PVA are a very good source of the phenol group and its attribute in the results also. Samples having 3%, 4% and 5% of gelatin showed 77%, 78% and 80% inhibition rates, respectively. This antioxidant activity rate of any food packaging is considered as ideal to enhance the shelf life of food items. As the concentration of gelation in increasing, the food packaging is becoming more antioxidant and beneficial for the food items.

Nanofiber mats having 5% gelatin ratio showed the optimum results and make it suitable for the application of food packing. The shelf life of food packaging has also been analyzed by using the 5% gelatin nanofiber mat to get the maximum results.

### 4.5. TGA Analysis

**Figure 5:**
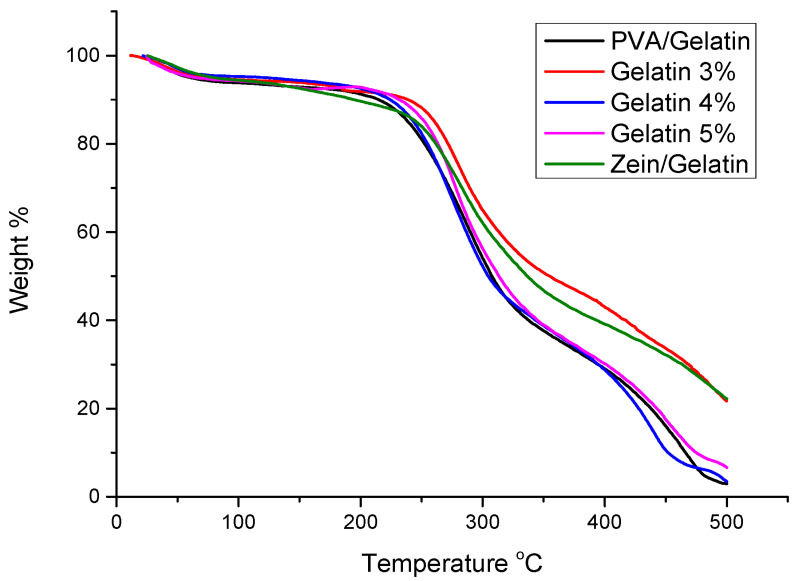
TGA analysis of PVA/Zein/Gelatin nanofibers.

Thermal stability of PVA/Zein/Gelatin nanofibers were investigated by TGA. Sometimes a food packaging has to undergo special temperature conditions, so thermal stability of the packaging materials up to a reasonable temperature limit is compulsory for the application of food packaging. It can easily be shown from Figure 5 that all samples show a similar trend in the temperature range of 25 °C to 100 °C which indicates the presence of volatile components in all samples. The onset temperature of PVA, zein and gelatin of all materials is up to 250 °C. Zein and gelatin indicates the major weight loss in the range of 275 °C and 250 °C, respectively, to 350 °C, while PVA shows the major loss in the range of 220 °C to 480 °C. All materials are thermally stable, and thermal stability of all materials strengthen each other and make the nanofiber thermally stable for the application of food packaging.

### 4.6. Mechanical Analysis

**Figure 6:**
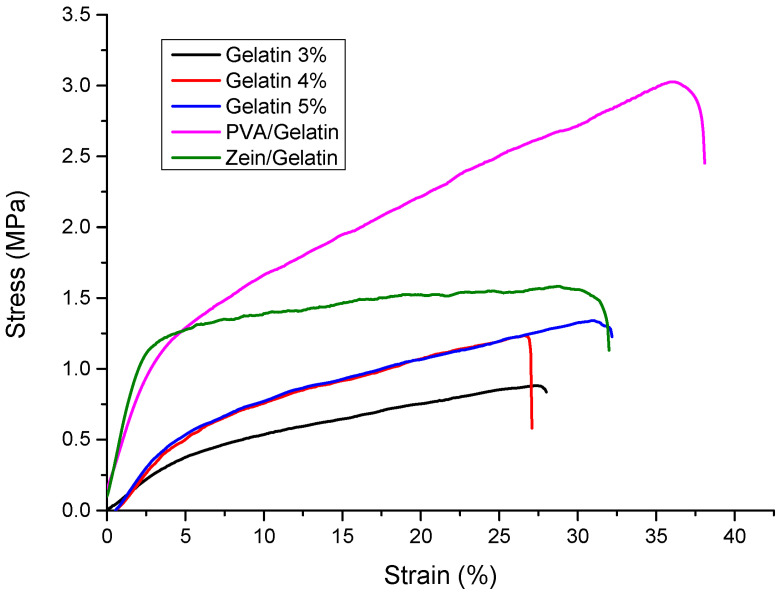
Stress strain curves of PVA/Zein/Gelatin samples.

Stress strain curves of PVA/Zein/Gelatin with different ratio of gelatin 3%, 4% and 5%, Zein/gelatin and PVA/Gelatin are shown in Figure 6. Mechanical strength is an important factor for the samples having an end application as active food packaging. The material should be able to bear stress and have excellent elongation at breaking for practical handling and packaging of food products. Zein itself is not mechanically strong enough but the presence of gelatin in addition increased the tensile strength. PVA is a mechanically stable polymer so its combination with gelatin also showed good mechanical strength. The sample with 3% gelatin showed a lower mechanical strength but as the concentration of gelatin increased the mechanical strength also increased. The sample with 5% ratio of gelatin showed excellent mechanical strength for the application of food packaging. It showed the good ability to bear stress along with excellent elongation at breaking which made it an appropriate material for active food packaging.

### 4.7. Water Contact Angle

**Figure 7:**
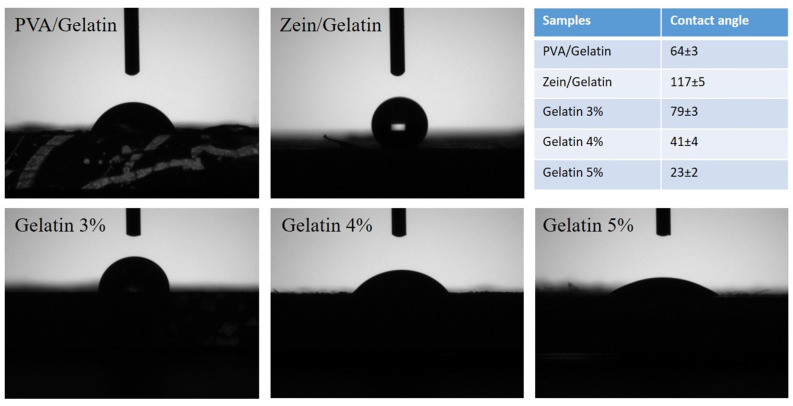
Water contact angle for PVA/Gelatin, Zein/Gelatin, and samples with varying concentration of gelatin.

Assessing the hydrophilic or hydrophobic behavior of materials is an important parameter in ascertaining their moisture vapor transport rate, bacterial growth, cell growth, environmental degradation, edging, and multiple characteristics of the final products. Un-crosslinked PVA is a highly hydrophilic polymer, and is soluble in water as well. Zein is a characteristically hydrophobic polymer, and partially soluble in water, however zein is soluble in a binary mixture of ethanol and water. From the contact angle measurements (Figure 7), electrospun nanofibers of zein/gelatin were found to be in a superhydrophobic range with an average contact angle of 117°, while PVA/gelatin nanofibers were hydrophilic due to the strong hydrophilicity of PVA. An average contact angle for PVA/gelatin was 64°. Water contact angles for electrospun tri-component nanofibers of PVA/zein/gelatin were also in the hydrophilic range. The contact angle for samples with gelatin 3%, gelatin 4%, and gelatin 5% were 79°, 41°, and 23°, respectively.

### 4.8. Antibacterial Activity for Bacillus and E. coli Bacteria

**Figure 8:**
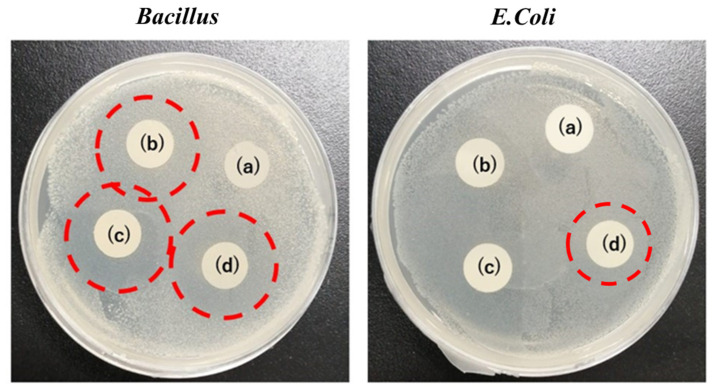
Antibacterial activity of *Bacillus* and *E. coli* bacteria (a) PVA/Gelatin, (b) Gelatin 3%, (c) Gelatin 4%, (d) Gelatin 5%.

The antibacterial test has been performed by using *Bacillus* and *E. coli* bacteria as shown in Figure 8 [38,39]. Samples having different concentration of gelation has been tested. Samples without gelatin did not show any antibacterial property for either *Bacillus* or *E. coli* bacteria. Samples b, c and d have 3%, 4% and 5% of gelatin respectively. As the concentration of gelatin increases, it was expected that antibacterial activity will also increase, however we could not observe much difference in any sample. However, OD_600_ measurements show that there was a slight decrease in the number of bacteria while increasing the gelatin content, but results are not satisfying enough to present. Increased shelf life might be because of some bacterial resistance as well but it can be concluded that the antioxidative properties played a major role rather than the antibacterial properties. Results from Figure 8 showed that the sample with a 5% concentration of gelatin is most suitable as a PVA/Zein/Gelatin nanofiber mat for food packaging application with a slight bacterial resistance (not antibacterial) against *Bacillus* and *E. coli.* Therefore, this sample could be helpful in maintaining the quality of food along with the enhanced shelf life of sweet potatoes, potatoes and Kimchi.

### 4.9. Shelf Life Test

#### 4.9.1. Shelf Life of Sweet Potatoes and Potatoes

**Figure 9:**
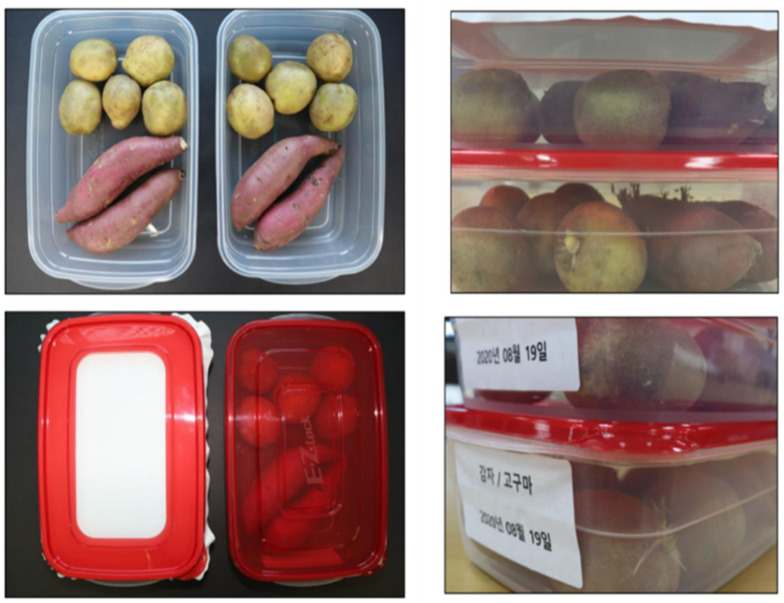
Shelf life analysis of sweet potatoes and potatoes for 50 days.

The shelf life of sweet potatoes and potatoes has been observed with and without food packaging. The nanofiber mat with 5% gelatin has been used to pack the sweet potatoes and potatoes. During the storage period, vegetables were kept under observation for 50 days. It can easily be seen from Figure 9 that both boxes were filled with the same quality of sweet potatoes and potatoes. One box was covered with a simple plastic lid while the other was covered with a nanofiber sheet. Both boxes were kept for observation under the same temperature and weather conditions. Sweet potatoes and potatoes covered with a nanofiber mat showed positive results and a delay in the ripping procedure. Sweet potatoes showed little sprouting while potatoes showed no abnormality at all. The box covered with a plastic lid showed sprouting with both sweet potatoes and potatoes, while fungus also started to grow on the surface of sweet potatoes and potatoes. It can easily be stated that the nanofiber mats can play the key role in extending the shelf life of sweet potatoes and potatoes.

#### 4.9.2. Shelf Life of Kimchi

**Figure 10:**
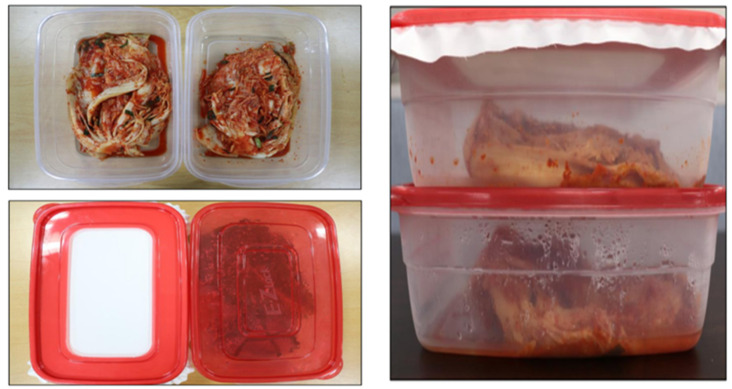
Shelf life analysis of Kimchi for 30 days.

Kimchi is one of the most popular dishes in Korean cuisine. Kimchi can be of different types, but for this particular test we used the basic type of cabbage Kimchi. Kimchi was packed in two boxes at the same time and boxes were kept under observation in the same conditions for 30 days. The nanofiber mat with 5% gelatin was used to pack the Kimchi. It was observed and can be seen easily from Figure 10 that Kimchi covered with a nanofiber mat is in good condition while Kimchi in another box is watery. It is clear from the results that the shelf life of Kimchi can be improved significantly by packaging it with a PVA/Zein/Gelation nanofiber mat.

Nanofibers have better breathability (MVTR and air permeability) and they help to maintain the humidity level suitable for food storage. Additionally, having antioxidant and antibacterial properties provides sufficient protection to the food inside the containers. It is expected that the antioxidant and bacterial-resistant nanofibrous food packaging will prolong the shelf life of different food items, especially those which have been passed through an extensive in-vivo storage test.

## 5. Conclusions

It is concluded from the above-mentioned results that the PVA/Zein/Gelatin-based active food packaging is novel and possesses excellent characteristics. Thermal properties were analyzed by TGA and showed that this food packaging is thermally stable. Results of the SEM analysis showed that all nanofiber mats had uniform morphology but the sample with a 5% gelatin ratio showed smooth morphology without any beads. Results of the antioxidant activity showed that all samples possessed very good antioxidant properties but the sample with a 5% gelatin ratio showed the maximum antioxidant activity, which means that it can be used as active food packaging. Antibacterial activities have been analyzed for both *Bacillus* and *E. coli* bacteria. PVA/Zein/Gelatin-based food packaging showed slight resistance against both *Bacillus* and *E. coli* bacteria. All characteristics showed that the sample with a 5% gelatin ratio is best to use as active food packaging. For this reason, the sample with a 5% gelatin ratio was analyzed to investigate the shelf life of sweet potatoes, potatoes and Kimchi. Sweet potatoes and potatoes were analyzed for 50 days, while Kimchi was kept under observation for 30 days. It was concluded that the novel food packaging can be used as active food packaging to increase the shelf life and can provide protection against bacteria.

## Data Availability

Data will be made available on request.
